# Association of TNF-α polymorphisms rs1800629 (−308G>A) and rs361525 (−238G>A) with type 2 diabetes mellitus in the Punjabi population of Pakistan

**DOI:** 10.3389/fendo.2025.1664411

**Published:** 2025-11-05

**Authors:** Muhammad Waqar, Tsai-Sung Tai, Abdul Qadeer, Umar Aziz, Mohammad Abohassan, Chien-Chin Chen, Sawar Khan

**Affiliations:** ^1^ Institute of Molecular Biology and Biotechnology, The University of Lahore, Lahore, Pakistan; ^2^ Division of Endocrinology and Metabolism, Department of Internal Medicine, Ditmanson Medical Foundation Chia-Yi Christian Hospital, Chiayi, Taiwan; ^3^ Department of Cell Biology, School of Life Sciences, Central South University, Changsha, China; ^4^ College of Animal Science and Technology, Northwest A&F University, Yangling, China; ^5^ Department of Clinical Laboratory Sciences, College of Applied Medical Sciences, King Khalid University, Abha, Saudi Arabia; ^6^ Health and Medical Researches Centre, King Khalid University, Abha, Saudi Arabia; ^7^ Department of Pathology, Ditmanson Medical Foundation Chia-Yi Christian Hospital, Chiayi, Taiwan; ^8^ Department of Cosmetic Science, Chia Nan University of Pharmacy and Science, Tainan, Taiwan; ^9^ Doctoral Program in Translational Medicine, National Chung Hsing University, Taichung, Taiwan; ^10^ Department of Biotechnology and Bioindustry Sciences, College of Bioscience and Biotechnology, National Cheng Kung University, Tainan, Taiwan

**Keywords:** type 2 diabetes mellitus, TNF-α promoter polymorphism, genetic association, Punjabi population, rs361525, rs1800629

## Abstract

**Background:**

The *TNF‐α* -promoter polymorphisms rs1800629 (−308G>A) and rs361525 (−238G>A) have been variably associated with type 2 diabetes mellitus (T2DM) risk in different populations. This study evaluated these two polymorphisms in a cohort of Punjabi individuals from Pakistan.

**Methods:**

A case–control study was conducted including 100 clinically diagnosed T2DM patients and 100 healthy controls. Genotyping of *TNF‐α* rs1800629 and rs361525 was performed using allele‐specific ARMS-PCR and validated by sequencing. Allele and genotype frequencies were compared between groups, odds ratios (ORs) and 95% confidence intervals (CIs) were calculated, and Hardy–Weinberg equilibrium was assessed.

**Results:**

The A alleles of rs1800629 and rs361525 were significantly more frequent in T2DM cases compared to controls (3.68% vs. 0.54%, OR = 6.779, p = 0.037 for rs1800629; 5.26% vs. 0.53%, OR = 9.684, p = 0.006 for rs361525). Fasting blood sugar level (FBS) of 150 ± 45 mg/dL was recorded in diabetic subjects. Multivariate and forest plot analyses supported the association of both variants with increased T2DM risk. Control group genotypes conformed to Hardy-Weinberg equilibrium, validating population stability.

**Conclusion:**

In this Punjabi cohort from Pakistan, the A alleles of TNF-α promoter polymorphisms rs1800629 and rs361525 were significantly more frequent in T2DM cases, indicating that these variants increase susceptibility to T2DM in this population.

## 1 Introduction

Type 2 diabetes mellitus (T2DM) represents a multifaceted metabolic disorder defined by persistent hyperglycemia arising from a combination of peripheral insulin resistance and inadequate compensatory insulin secretion (1, 2). Over the past several decades, T2DM has evolved into a global epidemic, with more than 450 million individuals affected worldwide and projections estimating upward of 700 million cases by 2045 (3, 4). The burden of disease is particularly acute in South Asia, where rapid urbanization, lifestyle transitions, and a high prevalence of genetic susceptibility converge to drive incidence rates that exceed global averages. In Pakistan, national health surveys report adult diabetes prevalence estimates varying between 14% and 20%, with many cases remaining undiagnosed due to limited screening and healthcare access (5, 6). The economic, social, and health-related consequences of T2DM in this region—ranging from microvascular and macrovascular complications to increased mortality—underscore the urgent need for improved risk stratification, pathophysiological understanding, and targeted prevention strategies (7, 8).

A growing body of evidence implicates chronic low-grade inflammation as a central mechanistic link between adiposity, insulin resistance, and β-cell dysfunction (9, 10). Tumor necrosis factor-alpha (*TNF-α*) is a multifunctional cytokine that regulates many physiological processes, including cell growth and differentiation, programmed cell death, lipid homeostasis, and blood coagulation. Dysregulated *TNF-α* signaling has been implicated in numerous conditions such as autoimmune disorders, insulin resistance, systemic rheumatic diseases, cancer, and severe manifestations of SARS-CoV-2 infection (11, 12). Among the proinflammatory mediators, *TNF‐α* has been recognized as a key effector molecule that disrupts insulin signaling pathways (10, 13). Originally characterized for its role in cachexia and immune regulation, *TNF‐α* impairs insulin action by inducing serine phosphorylation of insulin receptor substrate‐1 (IRS‐1), thereby attenuating subsequent downstream signaling through the phosphatidylinositol 3‐kinase (PI3K)–Akt axis (14–16). Moreover, *TNF‐α* can downregulate expression and translocation of the glucose transporter GLUT4 in adipocytes and myocytes, exacerbating hyperglycemia (17, 18). Circulating *TNF‐α* levels are elevated in individuals with obesity and T2DM and correlate positively with measures of insulin resistance, such as the homeostasis model assessment of insulin resistance (HOMA‐IR), further implicating *TNF‐α*–driven inflammation in the pathogenesis of metabolic disease (19).

Genetic variation within the *TNF‐α* locus may modulate individual differences in cytokine expression and, consequently, influence susceptibility to insulin resistance and T2DM. The *TNF‐α* gene is located within the class III region of the major histocompatibility complex on chromosome 6p21.3, an area characterized by extensive linkage disequilibrium and polymorphic diversity (20, 21). Within its proximal promoter region, two single‐nucleotide polymorphisms (SNPs)—rs1800629 (–308G>A) and rs361525 (–238G>A)—have attracted particular attention for their functional consequences (22). *In vitro* reporter assays demonstrate that the A alleles at these positions enhance basal and inducible transcriptional activity relative to the common G alleles, likely via altered binding of transcription factors such as AP‐2 and Sp1 (23). Consistent with these findings, peripheral blood mononuclear cells from A‐allele carriers produce higher levels of *TNF‐α* upon lipopolysaccharide stimulation, suggesting that these promoter variants act as expression quantitative trait loci (eQTLs) in immune cells.

Population‐based association studies of *TNF‐α* promoter polymorphisms and T2DM risk have yielded heterogeneous results. Early investigations in European cohorts yielded conflicting evidence, with some studies reporting modest risk increases (ORs ≈ 1.2–1.5) for the –308A allele, while others observed null or even protective associations (24). Meta‐analyses incorporating these data suggested potential publication bias and highlighted the importance of ethnicity, environmental exposures, and study design heterogeneity (25). In South Asian populations—comprising Pakistanis, Indians, Bangladeshis, and Sri Lankans—fewer studies have been conducted (26–28). Notably, there is a lack of genetic studies specifically focusing on the Punjabi population of Pakistan in relation to T2DM risk. Punjabi population constitutes a large and distinct ethnic group with unique genetic admixture and lifestyle patterns, including dietary preferences, physical activity levels, and sociocultural determinants that may interact with genetic predisposition to shape T2DM risk (29).

Given the functional relevance of these promoter polymorphisms and the paucity of data in Punjabi cohorts, the present study aimed to evaluate the association of *TNF‐α* rs1800629 (–308G>A) and rs361525 (–238G>A) with T2DM in a representative sample of Punjabi individuals from Pakistan. We employed allele‐specific amplification refractory mutation system PCR (ARMS‐PCR) for genotyping, with confirmatory Sanger sequencing to ensure accuracy. We compared allele and genotype distributions between T2DM patients and healthy controls, assessed conformity to Hardy–Weinberg equilibrium, and estimated odds ratios (ORs) and 95% confidence intervals (CIs) under various genetic models. Furthermore, we examined the relationship of these polymorphisms with fasting blood sugar (FBS) levels, to explore potential genotype–phenotype correlations that could shed light on mechanistic pathways. By elucidating the contribution of *TNF‐α* promoter variants to T2DM susceptibility and glycemic regulation in the Punjabi population, our study seeks to fill critical gaps in the understanding of inflammation‐driven genetic risk factors in South Asia. Identification of robust, population‐specific genetic markers holds promise for enhancing risk prediction, enabling early intervention, and ultimately informing precision medicine approaches tailored to regional genetic and environmental contexts. Moreover, our findings may have broader implications for unraveling the complex interplay between immune‐metabolic pathways and chronic disease, thereby contributing to global efforts to curb the escalating T2DM epidemic.

## 2 Methods

### 2.1 Study design and ethical approval

This case–control study was conducted between April 2022 and May 2023 in Lahore, Pakistan. Two hundred unrelated Punjabi individuals were enrolled: 100 patients with T2DM and 100 healthy controls. Sample size calculations were performed using QUANTO v1.2.4 software (University of Southern California, Los Angeles, CA, USA). The calculation incorporated the following parameters: an unmatched 1:1 case–control design, a log-additive genetic model, a risk allele frequency of 0.1381, disease prevalence of 0.20, α = 0.05 (two-sided), and 80% power. QUANTO estimated that 86 case–control pairs were required to achieve the desired power. T2DM cases were diagnosed at age ≥40 years with fasting plasma glucose ≥150 mg/dL. Exclusion criteria for both groups included type 1 diabetes, obesity, ocular disorders, autoimmune or chronic diseases. Further, the healthy control group does not include individuals with any form of hyperglycemia. Age (years), sex, weight (kg), and blood pressure (mmHg) were recorded for all participants, and each completed a structured questionnaire detailing medical history and lifestyle factors. Written informed consent was obtained from every participant, and the protocol was approved by the Ethical Review Committee of the Institute of Molecular Biology and Biotechnology, University of Lahore (Ref. IMBB/BBBC/22/30), in accordance with the Declaration of Helsinki.

### 2.2 Clinical and biochemical measurements

After an overnight fast, venous blood was drawn into EDTA and plain tubes. Plasma was separated by centrifugation (1,500 × g, 10 min) and stored at −80°C. FBS was measured via the glucose oxidase–peroxidase method on a semi-automated biochemistry analyzer (Mindray BA-88A). The FBS measurements were performed daily after blood collection. Body mass index (BMI) was calculated from measured weight and height, and blood pressure was taken using a calibrated sphygmomanometer.

### 2.3 DNA extraction

Genomic DNA was isolated from peripheral blood leukocytes by phenol–chloroform extraction. DNA purity and concentration were assessed spectrophotometrically (NanoDrop, Thermo Scientific) and by 1% agarose gel electrophoresis (Bio-Rad). Extracted DNA was stored at −20°C until genotyping.

### 2.4 Primer design and ARMS-PCR genotyping

The sequences flanking the *TNF-α* promoter SNPs rs1800629 (−308 G>A) and rs361525 (−238 G>A) were retrieved from NCBI dbSNP database. According to HGVS nomenclature, these variants correspond to NC_000006.12:g.31575254G>A and NC_000006.12:g.31575324G>A, respectively. Tetra-primer ARMS-PCR primers (**Supplementary Table S1**) were designed using SnapGene (https://www.snapgene.com/) and synthesized (50 nmol scale) by Macrogen (South Korea). Working stocks (10 pmol/µL) and primers (100 pmol/µL) were maintained at −20 °C. Each 25 µL PCR reaction was assembled to contain 1 µL of genomic DNA template, 2.5 µL of 10× Taq buffer, 2 µL of MgCl_2_ (to achieve a final concentration of 2 mM), 2 µL of a 2 mM dNTP mix, 0.3 µL of each of the inner primers (10 pmol/µL), 0.7 µL of each of the outer primers (10 pmol/µL), 0.125 µL of Taq DNA polymerase (5 U/µL), and 15.375 µL of nuclease-free water. Thermal cycling was performed on thermal cycler (Applied Biosystems) with an initial denaturation step at 95°C for 5 minutes, followed by 35 cycles of denaturation at 94°C for 40 seconds, annealing at 54°C for 1 minute, and extension at 72 °C for 1 minute. A final extension was carried out at 72°C for 10 minutes, after which the reactions were held at 4 °C. PCR products were resolved on 1% agarose gels stained with ethidium bromide and visualized under UV light. Allele-specific band sizes are given in **Supplementary Table S2**.

### 2.5 Sequencing validation and alignment

To confirm ARMS-PCR genotypes, 10% of samples representing each genotype were submitted for commercial sequencing (BTSeq™ technology, Celemics, Inc., South Korea). Raw chromatograms were inspected in SnapGene, and multiple sequence alignments were performed with CLUSTAL-W in BioEdit to verify the presence of G>A substitutions at –308 and –238.

### 2.6 Genotyping quality control and statistical analysis

Ten percent of all samples were re-genotyped in duplicate; concordance exceeded 99%. Overall call rates were > 98% for both SNPs. Genotype distributions in controls were tested for Hardy–Weinberg equilibrium using the χ² goodness-of-fit test (p > 0.05 denoting equilibrium). Statistical analyses were performed using SPSS v.21.0 (IBM Corp.). Allele and genotype frequencies were determined by direct counting. Continuous variables are presented as mean ± SD and compared by independent-samples t-test or one-way ANOVA. Categorical comparisons (genotype/allele frequencies) used χ² or Fisher’s exact tests. Odds ratios (ORs) and 95% confidence intervals (CIs) were calculated under additive and dominant genetic models. Associations between genotypes and FBS were evaluated by Kruskal–Wallis test with Dunn’s *post hoc* analysis. A two-tailed p-value <0.05 was considered statistically significant. Graphical representations, including raincloud plots and heat maps, were generated in R using the “ggplot2” and “ComplexHeatmap” packages.

## 3 Results

### 3.1 Demographic and clinical characteristics

A total of 200 participants were recruited for this study, comprising 100 patients with T2DM and 100 healthy controls. As summarized in **Table 1**, no significant difference was observed in sex distribution between diabetic and control groups (p = 0.45). However, statistically significant differences were evident in several clinical parameters. The mean age of diabetic subjects was significantly higher (60.5 ± 10.2 years) compared to controls (55.3 ± 12.1 years; p < 0.03). Diabetic individuals also exhibited higher mean body weight (85.3 ± 12.4 kg) than controls (72.1 ± 8.7 kg; p < 0.0002). Both systolic and diastolic blood pressures were significantly elevated in diabetic participants (p < 0.0001), and FBS was markedly higher in the diabetic group (150 ± 45 mg/dL) than in controls (95 ± 10 mg/dL; p < 0.0001).

**Table 1:** Demographic and clinical parameters of the participants.

Parameter	Diabetic (n=100)	Healthy (n=100)	P-value
Sex Ratio (M/F)	56/44	50/50	0.45
Age (years)	*60.5 ± 10.2; 35-85	55.3 ± 12.1; 22-78	<0.03
BMI (kg/m²)	27.9 ± 4.0; 22.6-36.4	23.5 ± 2.8; 17.3-27.7	<0.0001
Diastolic BP (mmHg)	85.4 ± 8.9; 70-110	75.3 ± 7.4; 60-95	<0.0001
Systolic BP (mmHg)	145.6 ± 15.2; 120-180	20.8 ± 10.3; 100-150	<0.0001
Fasting Blood Sugar (mg/dL)	150 ± 45; 90-300	95 ± 10; 70-120	<0.0001

*Values are shown as Mean ± SD; Range.

### 3.2 Genotyping validation

**Figure 1:**
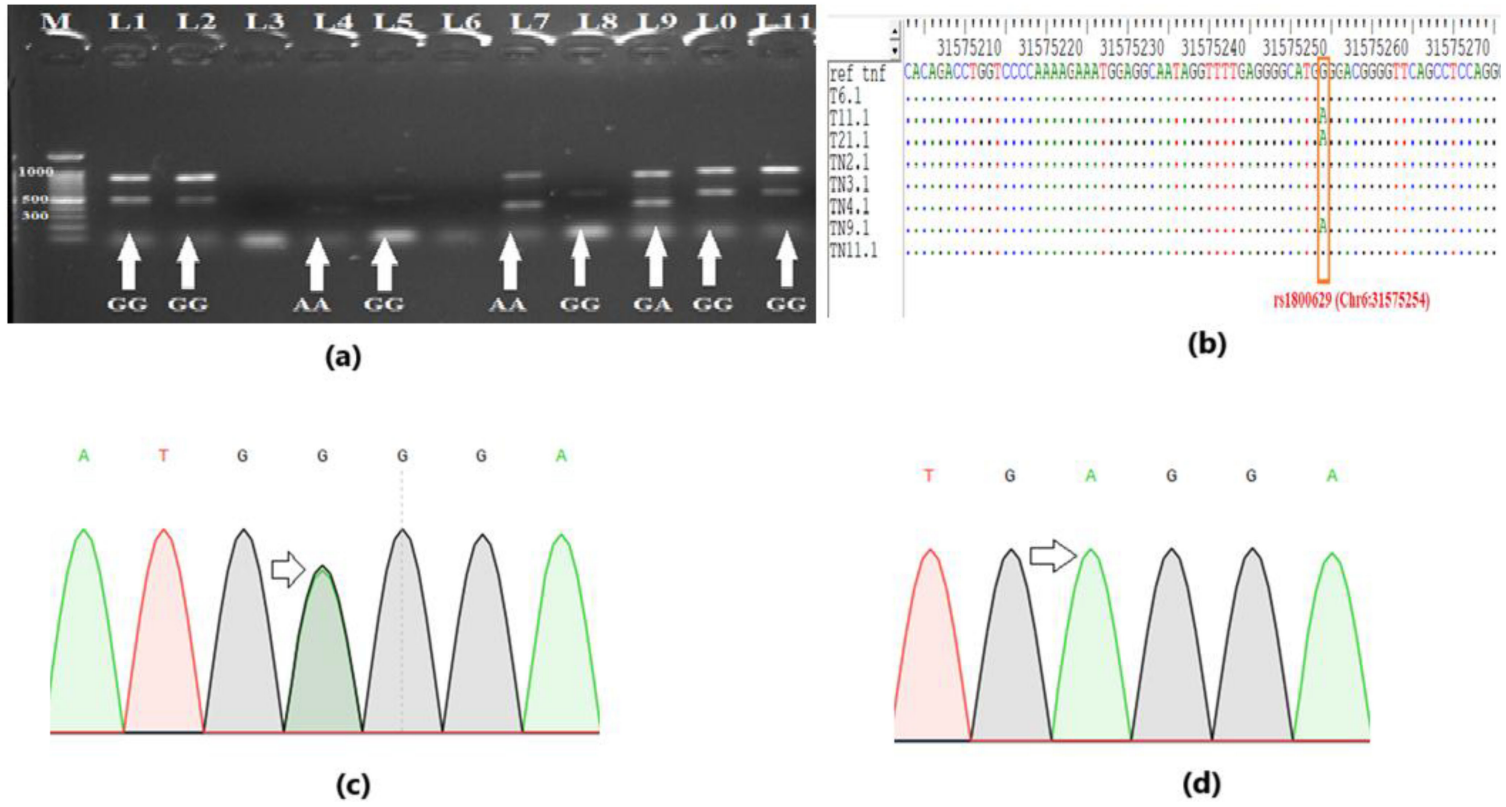
Genotyping for polymorphism. **(a)** Gel profile of Amplification-Refractory Mutation System-Polymerase Chain Reaction (ARMS-PCR), showing the representative genotypes. **(b)** DNA sequencing validates the genotyping results of ARMS-PCR. The single nucleotide polymorphism (SNP) “A” can be seen in multiple sequence alignment. **(c)** Sequencing chromatogram showing overlapped peak for heterozygous genotype “GA” **(d)** Sequencing chromatogram showing a single peak for homozygous genotype “AA”.

Genotypes for *TNF-α* polymorphisms rs1800629 (-308G>A) and rs361525 (-238G>A) were determined by ARMS-PCR and confirmed by Sanger sequencing (**Figure 1**). Representative gel images and sequencing chromatograms demonstrating the GG, GA, and AA genotypes are presented in **Figure 1**. The ARMS-PCR results showed clear amplification patterns for wild-type and mutant alleles (**Figure 1a**), which were fully concordant with sequencing chromatograms and alignments (**Figures 1b–d**), and successfully identified the “A” allele polymorphism.

### 3.3 Differential distribution of genotypes and alleles between case and control groups

**Figure 2:**
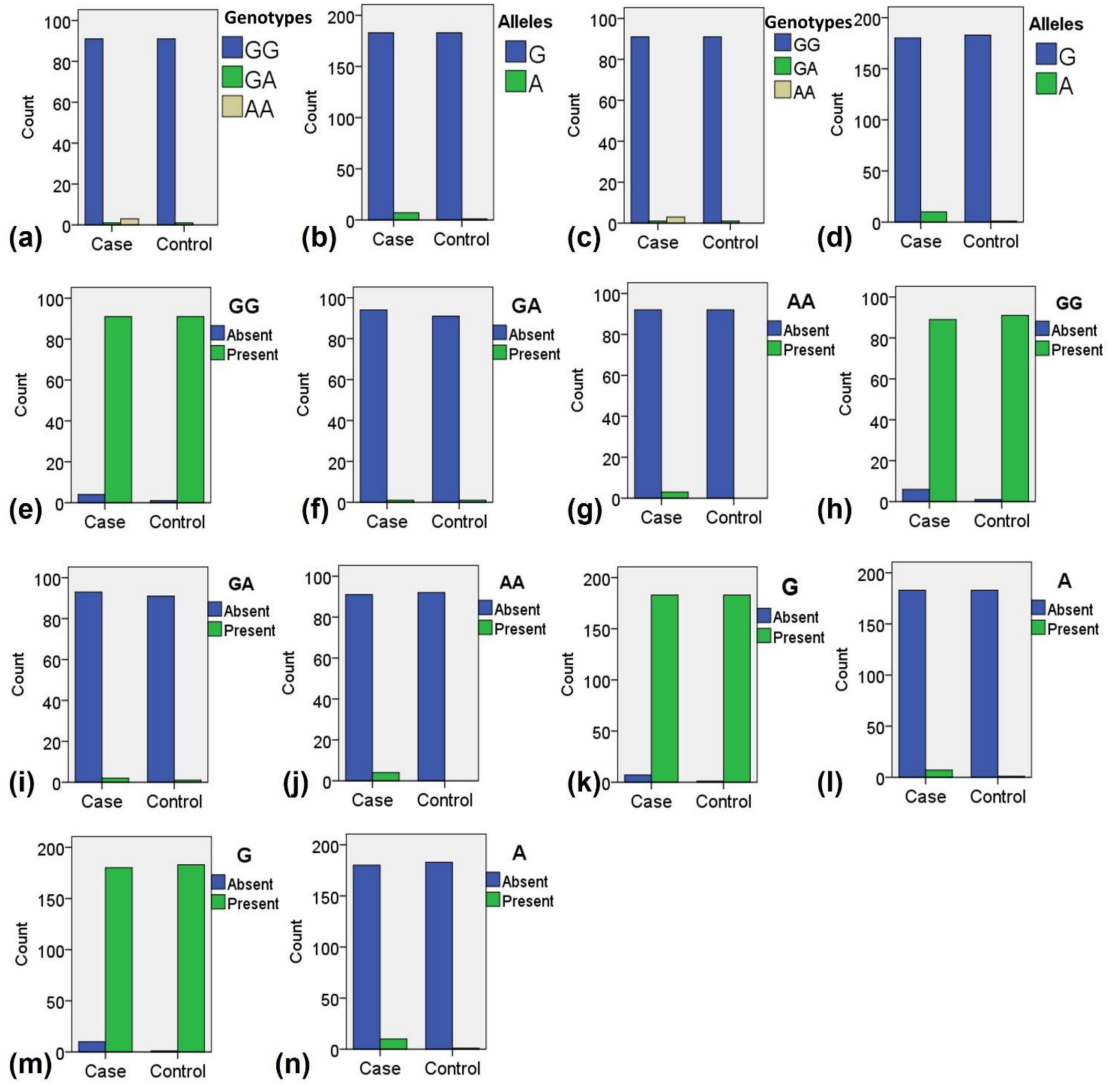
Distribution of genotypes and alleles among the case and control samples during the present study. **(a)** Overall distribution of all genotypes for polymorphism rs1800629. **(b)** Overall distribution of all alleles for polymorphism rs1800629. **(c)** Overall distribution of all genotypes for polymorphism rs361525. **(d)** Overall distribution of all alleles for polymorphism rs361525. **(e)** Distribution of normal genotype GG for polymorphism rs1800629. **(f)** Distribution of heterozygous mutant genotype GA for polymorphism rs1800629. **(g)** Distribution of homozygous mutant genotype AA for polymorphism rs1800629. **(h)** Distribution of normal genotype GG for polymorphism rs361525. **(i)** Distribution of heterozygous mutant genotype GA for polymorphism rs361525. **(j)** Distribution of homozygous mutant genotype AA for polymorphism rs361525. **(k)** Distribution of normal allele G for polymorphism rs1800629. **(i)** Distribution of mutant allele A for polymorphism rs1800629. **(m)** Distribution of normal allele G for polymorphism rs361525. **(n)** Distribution of normal allele A for polymorphism rs361525.

The final genotyping analysis included 92 samples from the control group and 95 samples from the T2DM group. To examine the distribution of genotypes and alleles between the case and control groups, we visualized the patterns associated with *TNF-α* polymorphisms rs1800629 and rs361525. The results revealed a clear difference in the genotypic and allelic distributions between the two groups (**Figures 2a, b**). For rs1800629, the overall genotypic distribution showed a predominance of the GG genotype in both groups, but the case group exhibited higher frequencies of heterozygous GA and homozygous mutant AA genotypes compared to the control group (**Figure 2a**). Similarly, allelic distribution indicated that while the G allele was predominant in both groups, the A allele was more prevalent in the case group than in the control group (**Figure 2b**).

A comparable trend was observed for rs361525. The GG genotype was more frequent in controls, while the GA and AA genotypes were notably higher in the case group (**Figure 2c**). Allelic analysis further supported this finding, as the G allele was dominant in controls, whereas the A allele was more abundant in the case group (**Figure 2d**). When analyzing individual genotype distributions, the normal GG genotype of rs1800629 was mostly present in controls (**Figure 2e**), while the homozygous AA genotypes were more frequently observed in cases (**Figure 2g**). For rs361525, the GG genotype was again predominant in controls (**Figure 2h**), whereas the AA genotypes were enriched in the case group (**Figure 2j**).

Regarding allele-specific distribution, the normal G allele of rs1800629 was predominantly observed in the control group (**Figure 2k**), whereas the mutant A allele was more frequently detected in the case group (**Figure 2l**). Similarly, for rs361525, the G allele was more common in the control group (**Figure 2m**), whereas the A allele was notably higher in the case group (**Figure 2n**). These findings suggest a differential distribution of *TNF-α* genotypes and alleles between the case and control groups, with a pronounced enrichment of mutant genotypes and alleles in the case group. The observed pattern supported further exploration into their potential association with disease susceptibility.

### 3.4 Association of rs1800629 and rs361525 with T2DM in Punjabi subjects

To investigate the association of *TNF-α* polymorphisms rs1800629 (−308G>A) and rs361525 (−238G>A) with T2DM, we compared genotype and allele frequencies between case and control groups (**Table 2**). For rs1800629, the wild-type GG genotype was predominant in both groups but was slightly less frequent in cases (95.78%) compared to controls (98.94%). The homozygous mutant AA genotype was observed exclusively in the case group (3.15%), while the heterozygous GA genotype was rare and evenly distributed across both groups. Although genotype frequencies did not show statistically significant differences (p > 0.05), the allele distribution revealed a significant pattern.

**Table 2:** Genotype and allele frequencies for *TNF-α* polymorphisms rs1800629 (-308G>A) and rs361525 (-238G>A) across the samples and their association with type 2 diabetes mellitus during present study.

Condition	Genotypes	T2DM *n* (%)	Control *n* (%)	Odds ratio	95% CI	*P=*value
Polymorphism *TNF-α* (-308G>A)						
Wild type Homozygous Heterozygous	GG AA GA Allele G A	91(95.78%) 3 (3.15%) 1 (1.07%) 183 (96.32%) 7 (3.68%)	91 (98.94%) 0 (0%) 1 (1.06%) 183 (99.46%) 1 (0.54%)	4.000 0.968 1.033 0.968 6.779	0.439-36.481 0.38-1.004 0.64-16.763 0.940-0.998 0.842-54.559	0.194 0.129 0.743 0.037
Polymorphism *TNF-α* (-238G>A)						
Wild type Homozygous Heterozygous	GG AA GA Allele G A	89 (93.69%) 4 (4.21%) 2 (2.1%) 180 (94.74%) 10 (5.26%)	91 (98.94%) 0 (0%) 1 (1.06%) 183 (99.47%) 1 (0.53%)	6.135 0.958 0.511 0.953 9.684	0.724-51.990 0.918-0.999 0.046-5.734 0.920-0.987 1.252-74.896	0.064 0.065 0.512 0.006

The 95% CI shows the 95% confidence interval range (upper and lower) of odds ratio, and the p value shows the significance of association in Chi-Square test.

The mutant A allele of rs1800629 was more frequent in cases (3.68%) than in controls (0.54%), while the G allele was more predominant in controls (99.46%) than in cases (96.32%). This difference was statistically significant (p = 0.037). Notably, individuals lacking the G allele may be at an increased risk of developing diabetes, with an odds ratio as high as 6.779 for individuals carrying A allele of this polymorphism. A strong significant association of allele A of rs1800629 (p = 0.037) with T2DM was observed in the Punjabi population.

Similarly, for rs361525, the GG genotype was more frequent in controls (98.94%) than in cases (93.69%), while the AA genotype appeared only in the case group (4.21%). The GA genotype was present at low frequency in both groups. Although genotype-level differences did not reach statistical significance (p = 0.064–0.512), allele frequency analysis showed that the A allele was more common among cases (5.26%) than controls (0.53%), with the G allele being more frequent in the control group. The odds ratio for individuals carrying A allele reached 9.684, and the difference was statistically significant (p = 0.006), suggesting a strong association of allele A of rs361525 with T2DM in this population.

These findings suggest that individuals lacking the protective G allele and carrying the mutant A allele of either rs1800629 or rs361525 may have an increased genetic susceptibility to T2DM in the Punjabi population.

### 3.5 Multivariate and Hardy-Weinberg analyses

**Figure 3:**
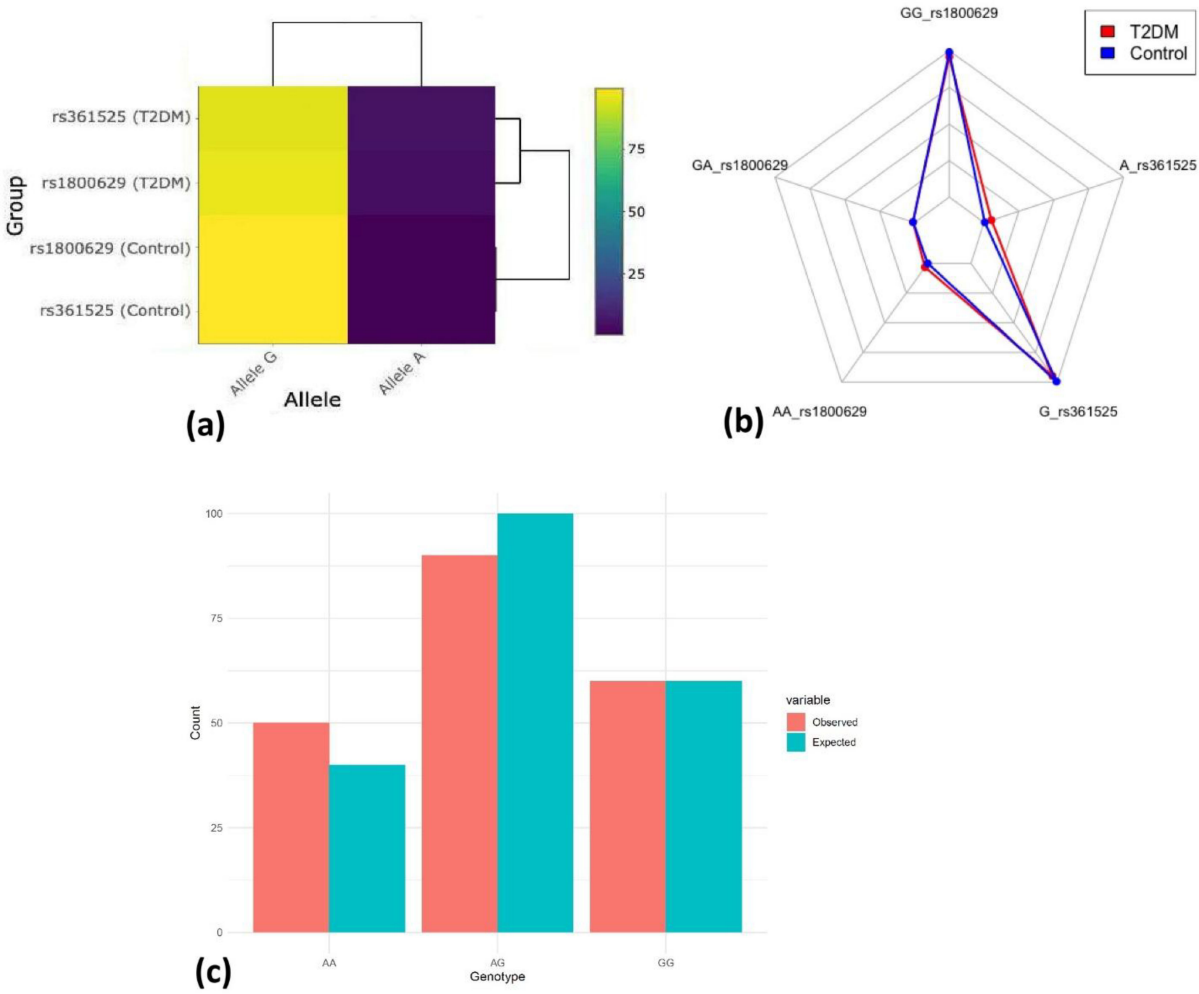
Multivariate visualization and neutrality. **(a)** Interactive allele frequency heat map. **(b)** Multi-genotype & allele radar chart. **(c)** Hardy–Weinberg equilibrium. Observed vs Expected genotypes (*p* = 0.174).

Multivariate visualization further supported these associations. The heat map (**Figure 3a**) and radar chart (**Figure 3b**) highlighted distinct patterns of allelic distribution and genotype frequency, respectively, between diabetic and control populations. Observed genotype frequencies conformed to Hardy-Weinberg equilibrium (HWE) expectations (*p* = 0.174) in the control group (**Figure 3c**), validating the genetic stability of the sample population, suggesting that there is no significant deviation from HWE.

**Figure 4:**
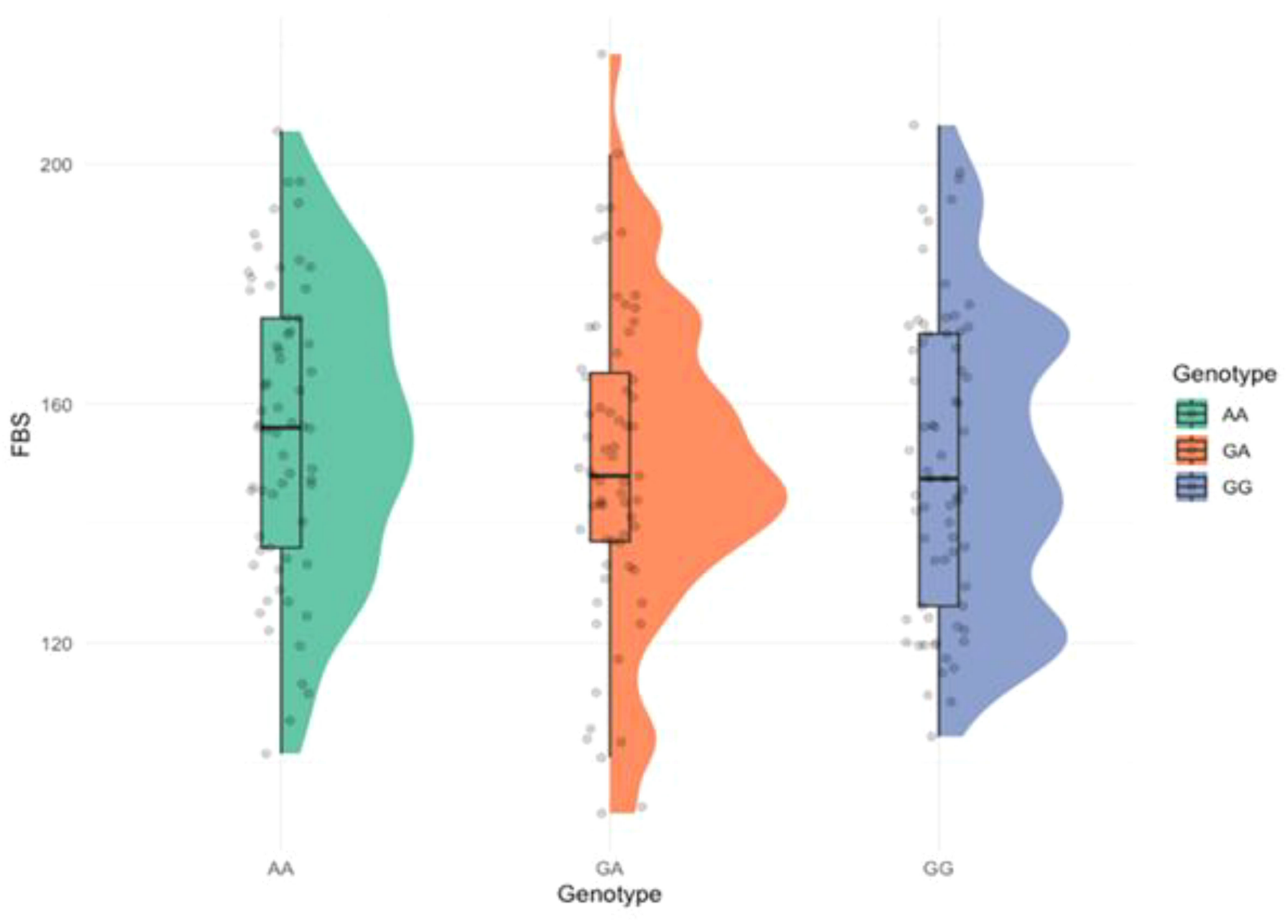
Raincloud plot showing the distribution of fasting blood sugar (FBS) across the genotypes. (*p* = 0.100).

The relationship between FBS levels and genotypic variation was explored using a raincloud plot (**Figure 4**). A noticeable trend was observed, where individuals carrying mutant genotypes tended to have higher FBS levels, particularly among those with the AA genotype, suggesting a genotype-phenotype correlation. However, the statistical significance was not observed (*p* = 0.100).

To further validate the observed genetic association, we conducted an odds ratio analysis using a forest plot for the rs361525 (−238G>A) polymorphism (**Supplementary Figure S1**). This analysis aimed to visually confirm whether the presence or absence of the protective G allele influenced the risk of developing T2DM. The results indicated that individuals in the Exposed2 group, those carrying the G allele, had significantly lower odds of developing T2DM, with an odds ratio of 0.225 (95% CI: 0.0827–0.612). This statistically significant negative association of the protective G allele reinforces our previous findings from the allele frequency analysis, which suggested that the A allele of rs361525 is significantly linked to an increased susceptibility to T2DM in the Punjabi population.

## 4 Discussion

Type 2 diabetes mellitus is a complex, multifactorial metabolic disorder that arises from the interplay of environmental triggers and genetic predisposition. Among the genes implicated in T2DM, *TNF-α*, a key pro-inflammatory cytokine, plays a critical role in insulin resistance and systemic inflammation (30–33). In this case–control study of a Punjabi cohort from Pakistan, we investigated the association of two promoter polymorphisms—rs1800629 (−308G>A) and rs361525 (−238G>A)—in the *TNF-α* gene with T2DM susceptibility. Our results demonstrate that the minor A alleles of both polymorphisms were significantly enriched in T2DM patients compared with healthy controls, and these alleles also showed an increasing trend with FBS, which may reach statistical significance in larger patient cohorts.

Allelic frequency analysis revealed that the A allele of rs1800629 was present in 3.69% of T2DM cases compared to 0.53% in controls (OR = 7.00, p = 0.037), while the A allele of rs361525 was observed in 5.26% of cases and only 0.53% of controls (OR = 10.167, p = 0.006). These significant associations indicate that individuals lacking the protective G allele at either locus may be at substantially higher risk of developing T2DM. These findings are consistent with reports from other ethnic groups, including Mexican, Indian, Malaysian and Han Chinese populations, where the A allele has been associated with enhanced disease risk (34–37).

We observed heterogeneity in BMI values between the patients and controls, with higher BMI values more prevalent in the diabetic group. Obesity is a well-recognized risk factor for the development of T2DM, and its interplay with hyperglycemia is primarily mediated through insulin resistance and chronic low-grade inflammation. Excess adiposity, particularly visceral fat, leads to adipocyte hypertrophy and macrophage infiltration into adipose tissue, which promotes the release of pro-inflammatory cytokines (9, 38). These inflammatory mediators impair insulin signaling pathways, thereby reducing glucose uptake and contributing to systemic insulin resistance (39, 40). Furthermore, obesity-induced dysregulation of adipokines, including leptin and adiponectin, exacerbates metabolic imbalance by favoring insulin resistance and hyperglycemia (41). This mechanistic link explains why individuals with higher BMI are at greater risk of developing T2DM, and why elevated BMI values are often observed among diabetic patients compared to non-diabetic controls. Importantly, even moderate increases in BMI have been associated with significant elevations in diabetes risk (42, 43), highlighting the strong dose–response relationship between excess weight and impaired glucose metabolism (44). Therefore, the heterogeneity in BMI distribution between patients and controls in our cohort likely reflects these underlying pathophysiological mechanisms, emphasizing the central role of obesity-driven insulin resistance and chronic inflammation in T2DM progression.

Functionally, *TNF‐α* is known to interfere with insulin signaling by promoting serine phosphorylation of IRS-1 and impairing the translocation of glucose transporter‐4 (GLUT-4) in adipocytes and muscle cells (45–48). Both the −308A and −238A alleles reside within key transcriptional regulatory motifs and have been shown to increase promoter activity, thereby enhancing *TNF-α* production, particularly under inflammatory conditions (49, 50). Elevated *TNF-α* levels, in turn, can exacerbate insulin resistance and contribute to the chronic low-grade inflammation observed in T2DM (19, 51). Thus, the presence of these promoter variants may represent a genetic predisposition toward increased systemic inflammation and metabolic dysregulation. Our findings extend this knowledge by demonstrating these associations in the Punjabi population of Pakistan, a group with distinct genetic and environmental backgrounds. Importantly, we observed that carriers of either A allele also had higher median FBS levels, further supporting a genotype-phenotype correlation. Raincloud plot analyses showed that individuals with the AA genotype, in particular, exhibited elevated FBS levels, suggesting a functional consequence of these polymorphisms beyond mere association. To validate our genetic findings, we employed multivariate visualizations and statistical modeling. Heat maps and radar charts clearly showed differential clustering of genotypes and alleles between cases and controls, highlighting their non-random distribution. Additionally, genotype frequencies in the control group conformed to Hardy-Weinberg equilibrium, validating the representativeness and genetic stability of the sample (52). These methods reinforced the robustness of our primary association results. A forest plot-based odds ratio analysis further confirmed that individuals carrying the risk-associated A allele of rs361525 had significantly higher odds of T2DM. Specifically, the odds ratio was 0.225 (95% CI: 0.0827–0.612) for individuals lacking the G allele, indicating a strong and statistically significant association. This visual confirmation strengthened the link between *TNF-α* promoter variants and increased diabetes risk.

While our findings are compelling, they must be interpreted in light of certain limitations. The relatively modest sample size (n=200) may limit statistical power for detecting genotype-level associations, especially for low-frequency homozygous mutant genotypes. Additionally, we did not assess serum *TNF-α* concentrations or downstream signaling markers, which would have provided functional validation of the genetic findings. Moreover, the study did not stratify results based on co-morbid conditions such as obesity or hypertension, which may modify genetic associations with T2DM. Despite these limitations, our results align with numerous earlier reports and meta-analyses supporting the association between *TNF-α* polymorphisms and T2DM (35, 53–55). Conflicting reports in the literature may be due to differences in population genetics, environmental exposures, or gene–gene interactions. Therefore, it is critical to conduct population-specific studies to accurately identify genetic risk markers.

Future research directions should include larger multicenter studies to confirm these associations and investigate potential gene–environment interactions, including lifestyle, dietary patterns, and physical activity. Functional assays measuring *TNF-α* mRNA or protein expression in carriers versus non-carriers of the G alleles would clarify the biological impact of these polymorphisms. Epigenetic profiling of the *TNF-α* promoter region and integration of these variants into polygenic risk scores could further improve risk prediction models for T2DM in South Asian populations.

## 5 Conclusion

The results of this study demonstrate a significant association between *TNF-α* promoter polymorphisms rs1800629 (−308G>A) and rs361525 (−238G>A) and increased susceptibility to T2DM in the Punjabi population of Pakistan. The A alleles of TNF-α promoter polymorphisms rs1800629 and rs361525 were significantly more frequent in T2DM cases and showed a positive trend with higher fasting glucose, suggesting increased susceptibility to T2DM that may be confirmed in larger cohorts. These polymorphisms may serve as promising genetic markers for early risk identification and underscore the pivotal role of inflammation in the pathophysiology of T2DM. Larger, more comprehensive studies are warranted to confirm these findings and translate them into clinical applications for personalized diabetes prevention and management.

## Data Availability

The original contributions presented in the study are included in the article/**Supplementary Material**. Further inquiries can be directed to the corresponding authors.
