# Supplementary material for: Association of TNF-α polymorphisms rs1800629 (−308G>A) and rs361525 (−238G>A) with type 2 diabetes mellitus in the Punjabi population of Pakistan
Source: Front Endocrinol (Lausanne). 2025 Nov 5;16:1664411. doi: 10.3389/fendo.2025.1664411 (PMC12626861; doi:10.3389/fendo.2025.1664411)
Supplement: Supplementary file 1 [file DataSheet1.docx]

Supplementary Material

# Supplementary Figures and Tables

| **Supplementary Table S1:** Primer sequences TNF-α (rs1800629 and rs361525) gene polymorphism | | | | |
| --- | --- | --- | --- | --- |
| **TNF-α Primer ID** | **Primer Sequence 5` to 3`** | **GC %** |  | **Tm** |
| **Outer Primers**  Outer Forward  Outer Reverse | GCATTATGAGTCTCCGGGTCAG  GTGGGTCAGTATGTGAGAGGAAG | 55 %  52% |  | 58 °C  57 °C |
| **Inner primer for rs1800629** | | | | |
| Inner Forward  Inner Reverse | CAATAGGTTTTGAGGGGCATGG  AGGCTGAACCCCGTCCT | 50%  65% |  | 58 °C  58°C |
| **Inner primer for rs361525** | | | | |
| Inner Forward  Inner Reverse | GACCCCCCTCGGAATCG  CCCATCCTCCCTGCTCT | 71%  65% |  | 58 °C  57 °C |

| **Supplementary Table S2:** Tetra- ARMS PCR product size information for TNF-α (rs1800629 and rs361525) | | | |
| --- | --- | --- | --- |
| **Primer** | **System** | **Allele** | **Amplicon** |
| **rs1800629** | | | |
| TNF-α IF  TNF-α IR  TNF-α OF  TNF-α OR | Forward inner primer  Reverse inner primer  Forward outer primer  Reverse outer primer | G  A  -  - | 396  477  830 |
| **rs361525** | | | |
| TNF-α IF  TNF-α IR  TNF-α OF  TNF-α OF | Forward inner primer  Reverse inner primer  Forward outer primer  Reverse outer primer | G  A  -  - | 445  402  830 |

## Supplementary Figures


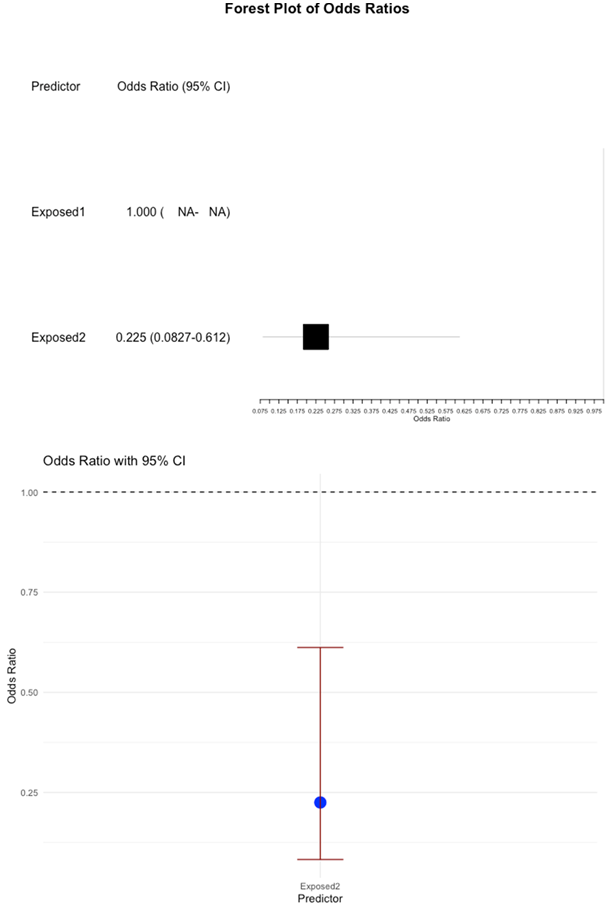


**Supplementary Figure S1.** Forest Plot of Odds Ratios. This plot displays the estimated odds ratios, The black square represents the point estimate of the OR, and the horizontal line indicates the 95% CI.
